# Editorial: Multiple Facets of H^+^-Pyrophosphatase and Related Enzymes

**DOI:** 10.3389/fpls.2016.01265

**Published:** 2016-08-29

**Authors:** Ali Ferjani, Masayoshi Maeshima

**Affiliations:** ^1^Department of Biology, Tokyo Gakugei UniversityKoganei, Japan; ^2^Laboratory of Cell Dynamics, Graduate School of Bioagricultural Sciences, Nagoya UniversityNagoya, Japan

**Keywords:** H^+^-pyrophosphatase, pyrophosphate, indolebutyric acid (IBA), Enoyl-CoA Hydratase 2, uncoupling H^+^-PPase variants, compensated cell enlargement, nitrogen metabolism, amine fungicides

Nearly 200 anabolic reactions liberate pyrophosphate (PPi), a byproduct of NTP hydrolysis (Kornberg, 1962; Maeshima, 2000; Heinonen, 2001; Ferjani et al., 2014a,b). In living cells, PPi must be hydrolyzed to orthophosphate by pyrophosphatase (PPase), because it suppresses the above reactions. PPases fall into two major classes, soluble PPases (sPPases) and membrane-bound H^+^-PPases (H^+^-PPases). In plants, vacuolar H^+^-PPase uses the energy released by PPi hydrolysis to acidify the vacuole, and its activity is particularly high in young tissues (Martinoia et al., 2007). Nevertheless, the physiological roles of PPases remain unclear due to severe phenotypes in loss-of-function mutants in various organisms (Ferjani et al., 2011 and references therein). Due to the importance of PPi homeostasis for life, this paper presents the most recent findings in this field and discusses the present situation along with future directions.

The *Arabidopsis* vacuolar H^+^-PPase AVP1 has been extensively characterized. Under physiological conditions, AVP1 is specifically localized to the tonoplast (Segami et al., 2014). It is also localized to the plasma membrane in sieve element companion cells, and upon overexpression in other cell types (Pizzio et al., 2015 and references therein; Khadilkar et al., 2016). In addition to its role in maintaining vacuolar pH, AVP1 controls auxin transport and, consequently, auxin-dependent development, based on *avp1-1* mutant analyses (Li et al., 2005). Recent studies using *fugu5* mutant alleles favored a role for AVP1 in PPi removal and did not confirm auxin-related phenotypes (Ferjani et al., 2011, 2014a,b; Bertoni, 2011). Genome sequencing revealed that a second T-DNA insertion in GNOM, which is required for auxin polar transport, is responsible for the *avp1-1* phenotype (Kriegel et al., 2015). Most importantly, Kriegel et al. (2015) further demonstrated that even plants lacking both tonoplast proton pumps (V-ATPase and AVP1) are viable and have significantly acidified vacuoles, highlighting for the first time the role of the TGN/EE-localized V-ATPase in vacuolar pH.

More recently, a unique approach adopted by Asaoka et al. allowed the independent evaluation of two AVP1 functions. First, they showed that overexpression of H^+^-PPase is not correlated with increased biomass (Asaoka et al., 2016), in agreement with Kriegel et al. (2015). Second, they produced a number of single-residue mutagenized H^+^-PPases, and successfully identified uncoupling mutant variants that retained PPi-hydrolyzing capacity, but failed to translocate protons. Interestingly, even such uncoupling variants complemented the developmental defects in *fugu5*, reinforcing the importance of PPi homeostasis in plant development. This timely contribution elegantly supports the importance of H^+^-PPase-mediated PPi hydrolysis *in planta*.

Recently, a curious relationship between nitrogen metabolism and PPi has also emerged. In fact, local cell death at the leaf petiole-blade junction was consistently observed in H^+^-PPase loss-of-function alleles upon growth on ammonia-free media (Fukuda et al.). Such phenotypes were totally suppressed when PPi was specifically removed by the yeast sPPase IPP1, by uncoupling H^+^-PPase variants, or by the simple addition of ammonium. Thus, it appears that high intracellular Pi levels somehow inhibit PPi hydrolysis, creating a cellular environment in which actively dividing cells can hardly cope.

In *Arabidopsis*, efficient usage of seed storage lipids is crucial for seedling establishment until the acquisition of photoautotrophy. Lack of H^+^-PPase in *fugu5* increases cytosolic PPi levels, partially reduces *de novo* sucrose synthesis, and inhibits cell division. In contrast, post-mitotic cell expansion in cotyledons is unusually enhanced, a phenotype called “compensation.” Therefore, it appears that PPi inhibits several cellular functions, including cell cycling, and it triggers compensated cell enlargement (CCE), whose mechanism remains elusive. Katano et al. reported that mutations in enoyl-CoA hydratase 2 (ECH2) significantly and specifically reduced CCE in the *fugu5* background. Together, defects in either H^+^-PPase or ECH2 reduce cell proliferation due to reduced seed storage lipid mobilization, but ECH2 alone likely promotes post-mitotic cell expansion in cotyledons, probably through the conversion of indolebutyric acid (IBA) to indole acetic acid (IAA). This provides a strong genetic and phenotypic basis to propose that the phytohormone auxin promotes CCE in *fugu5*, but not in other classes of compensation. ECH2 has a dual function, i.e., seed oil reserve mobilization and IBA-to-IAA conversion. Therefore, future studies using mutants with defects only in IBA-to-IAA conversion (Strader et al., 2010) should clarify the above issue.

Fungal pests are a major biological threat since they include the largest number of plant pathogens. While plant vacuoles have a dual set of proton pumps (V-ATPase and H^+^-PPase), fungi possess only V-ATPase. Amine fungicides are believed to inhibit postlanosterol sterol biosynthesis in fungi resulting in the accumulation of toxic abnormal sterols. Abnormal sterols affect the H^+^-pumping capacity of V-ATPases in fungi, and this has been proposed as a major determinant of fungicide action. Using yeast as a model fungus, Hernández et al. showed that amine fungicide treatment induced cell death by apoptosis, that apoptosis was concomitant with impaired H^+^-pumping capacity in vacuolar membrane vesicles dependent on vacuolar proteases, and that heterologous expression of *Arabidopsis* AVP1 in yeast cells increased resistance to amine fungicides. This paper challenged a long-standing issue in plant biology, and suggested that vacuolar H^+^-PPase is a major determinant of plant tolerance to amine fungicides.

Since the discovery of PPi in rat liver extracts in 1941 (see Cori et al., 1951), a large amount of work has aimed to uncover the biological roles of PPi-hydrolyzing enzymes. These discoveries do not herald the end of the H^+^-PPase road, which has become more complex. Now that there are genuine H^+^-PPase mutations, we should maintain a broad enough vision to understand H^+^-PPase contributions in a wide range of organisms, but simultaneously narrow enough to uncover its detailed molecular and physiological functions. H^+^-PPase overexpression is beneficial under stressful conditions (Gaxiola et al., 2016 and references therein), yet how stress tolerance is increased needs to be mechanistically elucidated. The four papers published on this research topic have deepened our understanding of plant vacuolar H^+^-PPase and identified new challenges that should be addressed in the future.

## Author contributions

AF and MM wrote and approved the final manuscript.

### Conflict of interest statement

The authors declare that the research was conducted in the absence of any commercial or financial relationships that could be construed as a potential conflict of interest.
